# Clinical leaders and providers’ perspectives on delivering medications for the treatment of opioid use disorder in Veteran Affairs’ facilities

**DOI:** 10.1186/s13722-021-00263-5

**Published:** 2021-09-06

**Authors:** Eric J. Hawkins, Anissa N. Danner, Carol A. Malte, Brittany E. Blanchard, Emily C. Williams, Hildi J. Hagedorn, Adam J. Gordon, Karen Drexler, Jennifer L. Burden, Jennifer Knoeppel, Aline Lott, George G. Sayre, Amanda M. Midboe, Andrew J. Saxon

**Affiliations:** 1 Health Services Research & Development (HSR&D) Seattle Center of Innovation for Veteran-Centered and Value-Driven Care, Veterans Affairs (VA) Puget Sound Health Care System, Seattle, WA USA; 2 Center of Excellence in Substance Addiction Treatment and Education, VA Puget Sound Health Care System Seattle Division (S116ATC), 1660 S. Columbian Way, Seattle, WA 98108 USA; 3 HSR&D Center for Care Delivery & Outcomes Research, Minneapolis VA Health Care System, Minneapolis, MN USA; 4 HSR&D Center of Innovation: Informatics, Decision-Enhancement, and Analytic Sciences Center, VA Salt Lake City Health Care System, Salt Lake City, UT USA; 5 VA Office of Mental Health and Suicide Prevention, Veterans Health Administration, Washington, DC USA; 6 Center for Innovation To Implementation, VA Palo Alto Health Care System, Menlo Park, CA USA; 7 Department of Health Services, University of Washington, Seattle, WA USA; 8 Department of Psychiatry and Behavioral Sciences, University of Washington School of Medicine, Seattle, WA USA; 9 Department of Psychiatry, University of Minnesota, Minneapolis, MN USA; 10 Program for Addiction Research, Clinical Care, Knowledge and Advocacy (PARCKA), Department of Internal Medicine, University of Utah School of Medicine, Salt Lake City, UT USA; 11 School of Medicine, Emory University, Atlanta, GA USA

**Keywords:** Opioid use disorder, Barriers, Facilitators, Buprenorphine, Primary care, Mental health, Implementation

## Abstract

**Background:**

Improving access to medication treatment of opioid use disorder (MOUD) is a national priority, yet common modifiable barriers (e.g., limited provider knowledge, negative beliefs about MOUD) often challenge implementation of MOUD delivery. To address these barriers, the VA launched a multifaceted implementation intervention focused on planning and educational strategies to increase MOUD delivery in 18 medical facilities. The purpose of this investigation was to determine if a multifaceted intervention approach to increase MOUD delivery changed providers’ perceptions about MOUD over the first year of implementation.

**Methods:**

Cross-disciplinary teams of clinic providers and leadership from primary care, pain, and mental health clinics at 18 VA medical facilities received invitations to complete an anonymous, electronic survey prior to intervention launch (baseline) and at 12- month follow-up. Responses were summarized using descriptive statistics, and changes over time were compared using regression models adjusted for gender and prescriber status, and clustered on facility. Responses to open-ended questions were thematically analyzed using a template analysis approach.

**Results:**

Survey response rates at baseline and follow-up were 57.1% (56/98) and 50.4% (61/121), respectively. At both time points, most respondents agreed that MOUD delivery is important (94.7 vs. 86.9%), lifesaving (92.8 vs. 88.5%) and evidence-based (85.2 vs. 89.5%). Over one-third (37.5%) viewed MOUD delivery as time-consuming, and only 53.7% affirmed that clinic providers wanted to prescribe MOUD at baseline; similar responses were seen at follow-up (34.5 and 52.4%, respectively). Respondents rated their knowledge about OUD, comfort discussing opioid use with patients, job satisfaction, ability to help patients with OUD, and support from colleagues favorably at both time points. Respondents’ ratings of MOUD delivery filling a gap in care were high but declined significantly from baseline to follow-up (85.7 vs. 73.7%, p < 0.04). Open-ended responses identified implementation barriers including lack of support to diagnose and treat OUD and lack of time.

**Conclusions:**

Although perceptions about MOUD generally were positive, targeted education and planning strategies did not improve providers’ and clinical leaders’ perceptions of MOUD over time. Strategies that improve leaders’ prioritization and support of MOUD and address time constraints related to delivering MOUD may increase access to MOUD in non-substance use treatment clinics.

**Supplementary Information:**

The online version contains supplementary material available at 10.1186/s13722-021-00263-5.

## Background

Between 1999 and 2018, the US opioid overdose epidemic claimed nearly 450,000 lives [1]. Although rates of opioid use and overdose deaths appeared to plateau in 2018, recent estimates of overdose deaths are again on the rise during the COVID-19 pandemic [2]. Ensuring individuals with opioid use disorder (OUD) have access to medication for OUD (MOUD) is critical, as formulations of methadone, buprenorphine, and extended-release naltrexone are associated with reduced mortality and/or opioid use [3–7]. Considering that less than 50% of individuals with OUD seek treatment for their OUD but may continue to seek medical care for other conditions, [8, 9] expanding access to MOUD in non-substance use disorder (SUD) specialty clinics or programs, such as primary care and other outpatient environments, has been recommended [10].

To expand access to MOUD, the US Department of Veteran Affairs (VA) launched the Stepped Care for Opioid Use Disorder Train the Trainer initiative (SCOUTT) [11]. SCOUTT aims to improve access to MOUD (i.e., buprenorphine and injectable naltrexone) across primary care, pain management, and mental health clinics using facility-based implementation teams at 18 VA facilities nationwide [12]. Through an external facilitation implementation framework based on prior studies [13], SCOUTT enabled local teams to use a population-based approach that promotes screening, assessment, and management of health conditions with the most effective, least resource-intensive intervention first, stepping up the intensity of care (e.g., SUD specialty care) as needed [14]. Several implementation strategies were used to support SCOUTT implementation, including planning (e.g., gather information, build buy-in, select strategies, initiate leadership, develop relationships), educating (e.g., develop materials, train, peer education, influence stakeholders), restructuring (e.g., revise professional roles, create new clinical teams) and managing quality strategies (e.g., audit/feedback, use advisory boards, organize clinician implementation team meetings) [15]. Thus, SCOUTT employed a multifaceted implementation approach which relied heavily on educating team members and planning for implementation.

The Consolidated Framework for Implementation Research (CFIR) is often used to evaluate the implementation of practice transformation initiatives, such as the SCOUTT initiative [16]. The CFIR, a meta-theoretical framework of factors associated with adoption of interventions, allows for assessment of complex multifaceted implementation strategies. The CFIR is organized into five major domains—intervention characteristics (e.g., advantage, complexity), outer setting (e.g., external policy, incentives), individual characteristics (e.g., attitudes, experience, knowledge and self-efficacy about the intervention), inner setting (e.g., the implementation context such as culture and climate), and process (e.g., the extent to which implementation is planned for, executed and evaluated) [17]. The model posits that successful adoption of an intervention (in this case stepped care for OUD) is a function of some combination of these five major domains.

### Purpose

The purpose of the current investigation was to determine whether the multifaceted approach used to implement SCOUTT yielded changes in commonly identified barriers and facilitators of MOUD prescribing. Previous work indicates barriers include beliefs that MOUD is too time-consuming (i.e., intervention characteristics), [12, 18, 19] limited provider knowledge about MOUD (i.e., individual characteristics), [12, 19, 20] and staff resistance (i.e., inner setting), [12, 18–20] while strong leadership facilitates MOUD adoption (i.e., inner setting) [21]. To maximize clinical utility and generalizability, we focused on intervention characteristics, inner setting, and individual characteristics. These represent common modifiable barriers and facilitators in the literature and potential targets for change in any clinic considering adding MOUD delivery to its services. Because the VA setting offers some unique facilitators (e.g., ease of access to SUD specialty care and consultants in a large integrated healthcare system) which may not generalize to non-VA settings, we did not examine outer setting (e.g., external policy, patient needs and resources). Further, because data from qualitative interviews with team members will be used to examine the process of implementation (e.g., engaging, execution, and reflection), this domain was not addressed by the survey.

While prior studies have examined primary care physicians’ attitudes regarding MOUD, to our knowledge, no previous work has examined perspectives about MOUD among providers across disciplines (i.e., physicians, nurses, psychologists, social workers, addiction therapists, pharmacists) longitudinally in non-SUD specialty clinics, including primary care, mental health, and pain management clinics. Further, this is the first study, to our knowledge, to examine whether a multifaceted implementation initiative to increase MOUD in non-SUD specialty care changes providers’ perceptions across relevant CFIR constructs. Given that multiple discrete strategies were utilized to provide education and decrease barriers, we hypothesized improvement in all areas over time. However, because changes to clinical workload (e.g., reduced panel sizes, protected time) were not addressed by SCOUTT implementation strategies, we do not expect changes in perceptions about MOUD being time-consuming.

## Methods

### Stepped Care for Opioid Use Disorder Train-the-Trainer (SCOUTT) initiative

Details regarding the national roll-out of the SCOUTT initiative in the VA have been previously reported [11, 22, 23]. Briefly, the SCOUTT initiative roll-out started with a request in May 2018 for all 18 regional VA network Directors to identify one facility and an interdisciplinary team of providers and clinical leaders at that facility to implement MOUD in at least one primary care, mental health, or pain management clinic in the first 6–8 months of implementation and one additional clinic at the same facility during the initial 12 months of implementation [11]. A team-based approach was emphasized for the SCOUTT initiative to potentially address insufficient time, a common barrier to provision of MOUD reported by primary care providers in the literature. The facility-level interdisciplinary teams (SCOUTT facility “implementation teams”) consisted of a prescriber to serve as a clinical champion, one additional prescriber (i.e., physician, nurse practitioner or physician assistant), one registered nurse, one therapist (i.e., a psychologist, social worker, or addiction therapist), and one clinical pharmacist. Teams were encouraged to include two prescribers to ensure coverage of buprenorphine prescribing when needed. Implementation team members received training in MOUD and stepped care at an in-person conference in August 2018. The assigned launch date for SCOUTT implementation was September 1, 2018. Each facility also was encouraged to assemble a team of SUD specialty care providers of similar disciplines to serve as consultants to implementation teams, as they implemented MOUD in their home clinic and trained providers in additional clinics at their facility. However, the focus of the current project is only on members of implementation teams. SUD specialty care consultants were surveyed separately. Attendees’ costs to attend the in-person training were reimbursed, but facilities received no additional financial support for SCOUTT participation.

The current project is part of a larger mixed-methods evaluation of SCOUTT implementation and is a partnership between the VA operations and researchers. Because SCOUTT is a quality improvement initiative, the VA Puget Sound Health Care System Institutional Review Board confirmed that the evaluation did not require human subjects’ approval.

### Sample

All providers on the implementation teams were eligible to participate. Eligible participants for the baseline survey were identified from implementation team rosters completed by each of the 18 participating facilities and confirmed with the clinical champion of each team. Prior to administering the follow-up survey, rosters were updated based on input from implementation teams to account for changes in team membership. The final sample sizes of eligible providers at baseline and follow-up were 98 and 121, respectively. The increase in the number of eligible providers from baseline to follow-up was largely due to teams assembling additional providers in the targeted primary care, mental health or pain clinic in the first year of implementation.

### Survey development

The survey instrument was developed based on review of published studies examining provider- and systems-level barriers and facilitators to providing MOUD and supplemented with items representing the CFIR domains inner setting, intervention characteristics, and individual characteristics to measure additional aspects of implementation. This process yielded a 43-item survey (Additional file 1), including demographics, that is organized into three domains (Additional file 2). To elicit narrative responses about barriers over year one, the open-ended question, *“What challenges have you faced in your role as a SCOUTT implementation clinic provider?”* was included in the follow-up survey. The survey was pilot tested by one primary care prescriber and two mental health providers (one social worker and one clinical pharmacist). Time for completion was measured (8–10 min), and questions that were unclear or redundant were identified. The lead author then interviewed each tester to identify questions that could be deleted, revised, or added, with responses used to iteratively revise the survey.

#### Intervention characteristics

A total of eight items were used to assess Intervention Characteristics. Three of the items were adapted from the Evidence subscale of the Organizational Readiness to Change Assessment (ORCA), [24] with “*Delivering medications (buprenorphine or naltrexone) to treat OUD in my clinic*” as the prompt. Three additional items were adapted from prior work by Wakeman and Barnett, [25] one was adapted from a survey developed by Netherland and colleagues, [26] and two were developed to address specific constructs of the domain.

#### Individual characteristics

To assess individual characteristics, we used the Drug and Drug Problems Perception Questionnaire (DDPPQ), which is comprised of 20 items across the following five subscales: Role Adequacy (e.g., provider knowledge; α = 0.94), Role Legitimacy (e.g., asking patients about opioid use; α = 0.89), Role Support (e.g., support available from colleagues; α = 0.78), Role-Specific Self-Esteem (e.g., professional ability to help; α = 0.69), and Job Satisfaction (e.g., satisfaction from working with opioid users; α = 0.80) [27]. “Drug” was replaced with “opioid” for all items, which are scored on a 1 (strongly agree) to 7 (strongly disagree) Likert scale. Lower scores indicate more favorable responses.

#### Inner setting

The ORCA Context subscale, which measures organizational readiness and aspects of clinic leadership, was used to assess inner setting with 19 items across five subscales (i.e., Leader Culture, Leadership Behavior, Measurement, Staff Culture, Opinion Leaders; Cronbach’s alpha = 0.85), with responses ranging from 1 (strongly disagree) to 5 (strongly agree) [24].

#### Survey distribution

All SCOUTT implementation team members received an anonymous electronic survey using an individualized e-mail with an embedded survey link in September 2018, which corresponds to the initial month of SCOUTT implementation, followed by three subsequent reminder emails sent at 1-week intervals. The 12-month follow-up survey was sent in November 2019 using similar procedures. Survey participation was voluntary, and participants had the option to not respond to any question. To ensure anonymity of participants, we did not use unique identifiers to link participants’ responses to sites or assess changes in participants’ responses from baseline to follow-up. No compensation was provided for survey completion.

### Data analysis

Descriptive statistics were used to summarize responses at baseline and 12-month follow-up, with means calculated for ORCA and DDPPQ summary scores, medians for continuous respondent characteristics, and percentages for categorical variables. Respondent characteristics at baseline and follow-up were compared using chi-square and Kruskal–Wallis tests for categorical and continuous variables, respectively. Similar response options were collapsed as recommended for categorical analyses to ensure each category contained adequate proportions (i.e. combined “strongly disagree,” with “disagree,” and “agree,” with “strongly agree”) [28]. Logistic regression models were used to compare the odds of responding strongly agree/agree at follow-up relative to baseline, adjusting for sex (female coded “1”; male coded “0”) and prescriber status (prescriber coded “1”; non-prescriber coded “0”) and clustered on regional network to account for correlated data. Linear regressions were used to compare differences in mean scores on ORCA and DDPPQ subscales over time, after adjusting for the same covariates. Narrative responses were reviewed verbatim and thematically analyzed by AD and EH using a template analysis approach to identify overarching concepts and themes [29]. Coding discrepancies were resolved through discussion and data review.

## Results

### Provider characteristics

Overall, 56 of 98 (57.1%) implementation team members responded to the baseline survey, representing clinics from all 18 regional networks. At follow-up, 61 of 121 (50.4%) members responded, representing 17 of the 18 regional networks. Table 1 shows demographic characteristics of respondents by time point, which were similar for both survey administrations. Approximately one-half of respondents were 25–44 years old, with over 50% identifying as female. Most providers identified as non-Hispanic, White (60–65%) and had practiced in their respective fields for more than 10 years. Physicians, pharmacists, and nurses were the most common disciplines. Among prescribers who completed the survey, 80.8% at baseline and 96.4% at follow-up had their DEA waiver to prescribe buprenorphine. Approximately, 61.9% and 74.1% of prescribers had prescribed buprenorphine to treat OUD at baseline and follow-up, respectively.

**Table 1:** Demographic Characteristics of Survey Respondents

	Baseline (*n* = 56)		Follow-up (*n* = 61)		
	*n*	%	*n*	%	*p*-value^a^
Survey Response per regional network, Mdn (Range)	3	1–7	3	0–8	
Age:					0.92
25–34	11	19.6	11	18.6	
35–44	20	35.7	18	30.5	
45–54	12	21.4	15	25.4	
55–64	9	16.1	12	20.3	
65 +	4	7.1	3	5.1	
Gender:					0.81
Female	33	58.9	34	56.7	
Male	23	41.1	26	43.3	
Total years of practice:					0.70
≤ 5	9	16.1	6	9.8	
5–10	14	25.0	19	31.1	
11–20	15	26.8	18	29.5	
20 +	18	32.1	18	29.5	
Race/Ethnicity:					0.52
Asian	9	16.4	7	11.7	
Black or African American	5	9.1	6	10.0	
Hispanic or Latino	4	7.3	1	1.7	
Multiple ethnicity / Other	4	7.3	7	11.7	
White/Caucasian	33	60.0	39	65.0	
Discipline:					0.58
Physician	18	32.1	25	41.7	
Nurse Practitioner	8	14.3	4	6.7	
Psychologist/Social Worker/Addictions Therapist	10	17.9	9	15.0	
Pharmacist	9	16.1	12	20.0	
Nurse	11	19.6	10	16.7	
Specialty:					0.98
Mental Health	21	37.5	25	41.0	
Pain Management	16	28.6	17	27.9	
Primary care/general internal medicine	16	28.6	16	26.2	
Multiple or Other	3	5.4	3	4.9	
Provider: Prescriber	26	46.4	28	45.9	0.95
DEA Waivered	21	80.8	27	96.4	0.07
Prescribed Buprenorphine for OUD past year	13	61.9	20	74.1	0.37

*Mdn* Median, *DEA Waivered* Drug Enforcement Agency Waiver to prescribe MOUD

*p < .05

^a^Based on Chi Square or Kruskal–Wallis tests for categorical and continuous variables, respectively

### Intervention characteristics

Table 2 compares responses to questions representing Intervention Characteristics. No statistically significant differences were found between responses at baseline and follow-up. With regard to delivering MOUD outside of SUD specialty care, most providers strongly agreed/agreed at baseline and follow-up that MOUD is supported by scientific evidence, consistent with clinical practices supported by VA patients and can be integrated into their clinic’s procedures and workflow. At baseline and follow-up most providers consistently agreed that delivery of MOUD is important and lifesaving. Few providers at baseline and follow-up reported MOUD as detracting from clinical responsibilities, being more dangerous than management of other chronic conditions, or risky in terms of patients diverting medications. However, slightly more than one-third of providers at baseline and follow-up reported MOUD delivery is time-consuming.

**Table 2:** Intervention characteristics and individual characteristics

	Baseline, %				Follow-up, %				
	*n*	Strongly disagree/Disagree	Neither Agree or Disagree	Strongly agree/Agree	*n*	Strongly disagree/Disagree	Neither Agree or Disagree	Strongly agree/Agree	*p*-value^d^
Intervention Characteristics									
Delivering medications (buprenorphine or naltrexone) to treat OUD in my clinic:									
Is important	54	1.8	0.0	94.7	57	1.6	4.9	86.9	0.16
Will save lives^b^	54	0.0	3.6	92.8	57	0.0	4.9	88.5	0.39
Is time consuming^b^	52	28.6	26.8	37.5	57	26.2	32.8	34.5	0.66
Detracts from my clinical responsibilities	53	50.7	19.6	14.3	56	52.5	24.6	14.8	0.87
Is more dangerous than management of other chronic conditions^b^	53	67.8	10.7	16.1	57	68.9	18.0	6.6	0.10
Is supported by randomized clinical trials or other scientific evidence^a^	54	1.9	13.0	85.2	57	1.8	8.8	89.5	0.60
Is consistent with clinical practices that have been accepted by VA patients^a^	54	3.7	11.1	85.2	57	3.5	19.3	78.2	0.20
Can be integrated into my clinic’s procedures and workflow^a^	54	7.1	14.3	75.0	56	8.2	14.8	68.9	0.64
Individual Characteristics									
Indicate your level of agreement or disagreement with the following statements:									
The risk of patients diverting these medications is too high^c^	54	64.8	22.2	13.0	58	56.9	32.8	10.3	0.76

Beliefs about Delivering Medication to Treat Opioid Use Disorder (OUD)

*OUD* opioid use disorder, *VA* Veteran’s Administration

*p < .05

^a^Item is from the Organizational Readiness to Change Assessment questionnaire

^b^Item based on Wakeman and Barnett, 2018.^19^

^c^Item adapted from Netherland, Botsko, Egan, et al., 2009.^20^

^d^Based on odds of responding Agree/Strongly Agree relative to other responses. Adjusted for gender and prescriber status and clustered on regional network

### Individual characteristics

Individual characteristics assessed by the DDPPQ are shown in Table 3. Providers’ ratings of knowledge about opioids (i.e., Role Adequacy), perceived right to ask about patient opioid use and related-consequences (i.e., Role Legitimacy), perceived support from colleagues in addressing issues with providing OUD treatment (i.e., Role Support), providers’ confidence in their professional ability and comfort with helping patients with an OUD ( i.e., Role-Related Self-Esteem), and finding work with opioid users rewarding (i.e., Job Satisfaction) were not statistically different across baseline and follow-up. Subscale means ranged from 2.0 to 2.9, which represent the “agree” response range.

**Table 3:** Individual characteristics

	Baseline (*n* = 56)			Follow-up (*n* = 61)		
	*n*	M (*SD*)		*n*	M (*SD*)	*p*-value*^a^
DDPPQ Subscale Score						
Role Adequacy	52	2.3 (1.4)		57	2.9 (1.7)	0.11
(e.g., I feel I have a working knowledge of opioids and opioid-related problems.)						
Role Legitimacy	52	2.0 (1.5)		57	2.6 (2.0)	0.12
(e.g., I feel I have the right to ask patients questions about their opioid use when necessary.)						
Role Support	50	2.1 (1.4)		54	2.6 (1.6)	0.20
(e.g., If I felt the need, I could easily find someone who would be able to help me formulate the best approach for an opioid user.)						
Self Esteem	51	2.2 (1.3)		54	2.6 (1.4)	0.09
(e.g., I often feel uncomfortable when working with opioid users. – reversed)						
Job Satisfaction	51	2.3 (1.4)		54	2.5 (1.6)	0.63
(e.g., In general, it is rewarding to work with opioid users.)						

Adapted Drug and Drug Problems Perceptions Questionnaire (DDPPQ) Subscale Scores

*DDPPQ* Drug and Drug Problems Perceptions Questionnaire, *M* = mean; *SD* standard deviation, DDPPQ responses are scaled from 1 to 7, with 1 indicating more favorable

responses. Example items from each subscale provided below means

*p < .05

^a^Based on estimated mean differences in scores at baseline and follow-up. Adjusted for gender and prescriber status and clustered on regional network

### Inner setting

Most providers consistently endorsed MOUD delivery as compatible with the care provided in their clinics across both time points (Table 4). While most also agreed that MOUD fills an important gap in care, ratings decreased significantly from 85.7% at baseline to 73.7% at follow-up. Further, only slightly more than half of providers agreed that providers in their clinics wanted to prescribe MOUD at baseline, with no improvement reported at follow-up.

**Table 4:** Inner setting

		Baseline, %				Follow-up, %			
	*n*	Strongly disagree/ Disagree	Neither Agree or Disagree	Strongly agree/Agree	*n*	Strongly disagree/ Disagree	Neither Agree or Disagree	Strongly agree/Agree	*p*-value*^b^
Delivering of medications (buprenorphine or naltrexone) to treat OUD in my clinic:									
Is compatible with the care provided by my clinic^a^	54	5.4	12.5	76.7	57	6.6	11.5	75.4	0.88
Fills an important gap in the care my clinic provides^a^	54	1.8	8.9	85.7	57	4.9	14.8	73.7	0.04*
Indicate your level of agreement or disagreement with the following statement:									
Providers in my clinic want to prescribe buprenorphine or naltrexone	54	26.0	20.4	53.7	57	22.8	24.6	52.6	0.94

Beliefs about delivering medication to treat opioid use disorder (OUD)

*OUD* opioid use disorder

*p < .05

^a^Item is from the Organizational Readiness to Change Assessment questionnaire

^b^Based on odds of responding Agree/Strongly Agree relative to other responses. Adjusted for gender and prescriber status and clustered on regional network

Subscale mean scores representing clinic Leader Culture and Leadership Behavior were the lowest among the five context subscales and did not improve across survey administrations (Table 5). Overall, respondents had mostly neutral ratings of leadership’s promotion of positive organizational culture and demonstration of positive leadership practices. At baseline, the Measurement subscale mean score also reflected a neutral rating of leadership setting goals, tracking, and communicating performance to staff, with ratings at follow-up reflecting no improvement. The Staff Culture and Opinion Leaders subscale scores changed little, though reflected favorable ratings of staff and opinion leaders’ willingness to innovate, improve, and change clinical practices to promote effective care.

**Table 5:** Inner setting

	Baseline (*n* = 56)		Follow-up (*n* = 61)		
	*n*	M (*SD*)	*n*	M (*SD*)	*p*-value^*a^
Average ORCA score					
Leader Culture	49	3.6 (1.1)	49	3.3 (1.1)	0.21
Leader Behavior	48	3.6 (1.0)	49	3.3 (1.0)	0.27
Measurement	48	3.8 (0.9)	50	3.5 (1.0)	0.12
Staff Culture	50	4.0 (0.9)	50	4.0 (0.7)	0.99
Opinion Leaders	48	3.9 (0.7)	48	4.1 (0.7)	0.08

Adapted Organizational Readiness to Change Assessment (ORCA) subscale scores

*ORCA* Organizational Readiness to Change Assessment; ORCA responses

range from 1 to 5, with 5 indicating more favorable responses. *M* = mean; *SD* standard deviation

*p < .05

^a^*p*-value derived from estimated mean differences in scores at baseline and follow-up

Adjusted for gender and prescriber status and clustered on regional network

### Self-reported perceived barriers

Several barriers reflecting organizational climate were identified from (*n* = 38) responses to the open-ended question regarding challenges in SCOUTT implementation over the first year. The most common barriers reported included lack of support to diagnose and treat OUD (*n* = 9; 23.7%), lack of protected time to implement MOUD (*n* = 9; 23.7%), and delays caused by issues with credentialing and privileging waivered prescribers (*n* = 7; 18.4%). Lack of provider knowledge about OUD (*n* = 3; 7.9%) and coordination and/or communication with other services (*n* = 3; 7.9%) were less frequently reported.

## Discussion

This is the first study, to our knowledge, to assess changes over time in commonly-reported barriers to providing MOUD associated with a large scale implementation of MOUD delivery in non-SUD specialty VA outpatient settings [12]. Contrary to our hypothesis, improvement was not shown in ratings of most implementation-related factors, despite SCOUTT consisting of multiple discrete implementation strategies. Further, respondents’ lower ratings on MOUD filling an important gap in care at follow-up relative to baseline was the only statistically significant difference between ratings. Consistent with our hypothesis, we found no changes in perceptions of MOUD as time-consuming. Provider-reported barriers included lack of support in diagnosing and treating OUD, time needed for MOUD, and delays in credentialing waivered prescribers. Among provider ratings, leadership behavior and culture and measurement were the lowest scored among Inner Setting CFIR constructs.

Despite the lack of differences from baseline to follow-up in providers’ perceptions of commonly -cited barriers to MOUD provision, findings from the current study, as guided by the CFIR, have implications for further implementation efforts to ensure that patients with OUD receive low-barrier, effective treatment. Below we summarize implications of findings with respect to the CFIR domains of interest.

### Intervention characteristics

Similar to findings from prior studies, providers had positive and stable views on the evidence, importance, and life-saving impact of MOUD [30]. In contrast to prior studies, few providers had concerns about patients diverting medications to treat OUD or about provision of MOUD detracting from or competing with other clinical duties [31, 32]. Further, providers’ views on these factors were consistent across survey administrations, suggesting that their experiences implementing MOUD did little to diminish the positive views reported at baseline.

Despite the SCOUTT initiative’s emphasis on assembling a team to deliver MOUD care, 1 in 3 providers had concerns about the time required to deliver MOUD, a barrier often reported by primary care providers in the literature [12, 19, 20, 30, 33]. Providers’ persistent views of the time demands of delivering MOUD suggest that increased experience with the intervention does not necessarily promote more efficient delivery, consistent with previous work [33]. Allowing for longer and/or more frequent visits, reducing caseloads of providers delivering MOUD, or adding case managers to teams may be necessary to address this barrier [30, 34]. It is also possible that participants endorsing this view were from teams that did not function cohesively or collaborate with each other to provide efficient care. More work is needed to understand if high functioning teams were able to address the time barrier often reported by providers delivering MOUD care.

Providers also reported delays in completion of credentialing and privileging processes required by each facility. As indicated on Table 1, the percentage of DEA waivered providers increased from 81% at baseline to 96% at follow-up; however, timely completion of these processes was among the most common barriers providers chose to report. Regulatory steps needed to obtain a waiver to prescribe buprenorphine are often identified as a barrier, with some advocating for eliminating this regulation [34, 35]. Perhaps, because waiver trainings were among the implementation strategies deployed, obtaining an x-waiver was not an identified barrier – rather, it is facility-specific policies on credentialing and privileging that delayed providers from delivering MOUD. Regardless, policy context strategies (i.e., one of two implementation strategy domains not directly addressed by SCOUTT) may be necessary. For example, standardizing credentialing and privileging across VA facilities may reduce delays in MOUD delivery. Because facilities are part of the VA healthcare system and expected to adhere the same clinical policies and guidelines, differences in facility-level prescribing policies were not anticipated. Assessing outer setting components that may influence successful implementation will be critical in future phases of the SCOUTT initiative.

Based on barriers of time and credentialing and privileging processes in the VA identified in part through interactions with SCOUTT teams, shortly after the initial year of SCOUTT, the VA distributed recommendations encouraging facilities to consider incentives (e.g., reduced panel sizes, financial remuneration) to promote the quality and timeliness of MOUD [36]. The recommendations also suggested eliminating credentialing and privileging barriers to MOUD implementation and affirmed that MOUD can be prescribed in all clinical environments. In addition, there have been calls to deregulate prescribing of buprenorphine for OUD to eliminate training requirements, X-waiver applications, and DEA audits, which deter prescribers from offering MOUD [34, 35]. Effective April 28, 2021, prescribers may apply for an X-waiver without completing training requirements that will allow them to prescribe buprenorphine to up to 30 patients [37].

### Individual characteristics

The literature is sparse regarding job satisfaction, perceived support, and job-related self-esteem of providers delivering MOUD, and what is available is limited to primary care prescribers [30, 33, 38, 39]. Consistent with a prior study, our respondents from multiple disciplines reported high satisfaction with treating patients with OUD [38]. Further, providers reported positive views of their effectiveness and comfort working with patients who use opioids, as well as their knowledge of opioid use and related consequences, and ability to access support from colleagues. Although prior work has reported lack of prescriber/staff knowledge as a barrier to MOUD delivery [13, 19, 20, 26, 40, 41], respondents to the survey reported high ratings of OUD knowledge at baseline. This suggests that providers received adequate training prior to their involvement in SCOUTT and/or the early SCOUTT trainings provided them with knowledge needed to deliver MOUD. Alternatively, it may be that implementation teams at facilities were selected by regional network leadership based on their baseline knowledge of and experience with delivering MOUD. Contrary to expectations, knowledge ratings did not improve over time, despite several strategies focused on educating providers (e.g., monthly virtual educational trainings, community of practice meetings, access to consultation with local experts). Given the high mean score at baseline, the lack of improvement in knowledge may be due to a ceiling effect. Most of the prescribers who completed the survey at baseline had their DEA waiver to prescribe buprenorphine and over one-half had prescribed it to treat OUD in the 12 months prior to SCOUTT implementation, suggesting a knowledgeable and relatively experienced group of prescribers. Nonetheless, the multi-strategy intervention used in SCOUTT was sufficient for maintaining but not improving positive individual characteristics.

### Inner setting

Providers endorsed MOUD as compatible with care delivered in their clinics and filling an important gap in care, though agreement with the latter question decreased significantly from baseline to follow-up. Given providers’ strong agreement with the scientific evidence and lifesaving potential of MOUD, this finding is likely not about changing views on the merit, importance, and potential impact of this treatment. Speculatively, it may reflect providers’ perception of low patient demand for MOUD in their clinics, changing perspectives on where care should be delivered, and/or experiencing MOUD delivery as more difficult and complex than anticipated [32, 33, 42].

Our finding that nearly one-half of respondents view their colleagues as lacking motivation or interest to deliver MOUD is a common finding in the literature [12, 30]. However, while ratings were stable across our study, previous qualitative longitudinal work has found that motivation to prescribe MOUD decreases over time [33]. One interpretation is that SCOUTT strategies were sufficient to maintain but not increase motivation from baseline. Financial strategies, which were not used in this project, such as prescriber-directed incentives to obtain an x-waiver and/or prescribe buprenorphine may encourage those with little interest to deliver MOUD [15, 43]. Physician-directed incentives have been associated with increases in physicians completing x-waiver training and the proportion of buprenorphine prescribing among clinical encounters involving an OUD [44].

Respondents’ mostly neutral ratings of leadership readiness to adopt new practices, promote team building, and promote communication persisted over time. Although prevalent in the broader implementation literature, few studies have identified leadership-related barriers in implementation of MOUD in outpatient settings. Among those studies, lack of leadership support was identified as a barrier to MOUD in VA residential facilities and community-based primary care, and lack of leadership support persisted but decreased over implementation phases in a primary care study [33, 45]. As ratings of leadership culture and behaviors were among the lowest of leadership ratings, it may be that the clinic leaders who implementation teams report to (e.g., chief of service or program lead) are influencing team dynamics and contributing to the stability, rather than improvement, of scores over time. Further, the dynamic between top clinical leaders and those at the team and/or clinic-level may have led to the provider-reported barrier of lack of support to diagnose and treat OUD [46]. This finding suggests implementation strategies directly targeting leaders may be necessary to improve implementation of stepped care for OUD. Additional work is needed to further understand how top clinical leaders influence providers' perception of support for MOUD and how leaders' involvement and support of MOUD delivery can be enhanced. Planning strategies that build buy-in from top leaders or involve executive boards with organizational support to prioritize change may influence clinical leaders’ willingness to promote and support MOUD delivery [15]. Further, providing opportunities for clinic-level leaders to communicate with top leaders about the types of support that would be most impactful in supporting providers’ commitment to MOUD implementation may also improve implementation effectiveness. Financial incentives for clinical leaders also may need to be considered to encourage leaders to prioritize provider-wide adoption and sustainment of MOUD delivery.

Because the VA is an integrated health care system with specialty substance use and mental health disorder services with national treatment guidelines, we considered components of the outer setting (e.g., external policies, incentives) to be less important to the SCOUTT initiative and did not include items related to this domain on the survey. However, several findings highlight the importance of this domain to the implementation of MOUD in the VA. The observed variation in the credentialing and privileging policies of x-waivered prescribers across participating facilities indicates that practices and policies can be influenced at the facility-level; changes in national policies and/or mandates may be needed to support implementation. Further, additional funding at the national-level may be needed to support financial incentives to encourage providers to adopt this new practice and facility leaders to commit adequate resources to spread and sustain MOUD at their facilities. As there are possibly other strategies at the external and/or VA organization level that are important to implementation, the lack of specific survey items to assess the outer setting is a limitation of this investigation. Further, implementation processes such as planning, executing plans and evaluating progress are all important components of implementation that were not examined by the survey, and thus represent another limitation of the current investigation. However, they will be addressed by other data collection approaches.

### Limitations

Our project has several limitations in addition to those noted above. Although data were obtained from implementation teams from across 18 VA medical centers, these findings may not generalize to other VA or non-VA settings. Because regional leaders could select the implementation clinic location and team members, results may be biased in an unquantifiable manner. This may have been compounded by one regional network not responding to the follow-up survey. The sample size was too small to examine differences by clinic type or prescriber status, and the survey response rate of 50% is low, increasing likelihood of further response bias. There were changes in team memberships between baseline and follow-up, and we do not know how representative responses were of the entire SCOUTT team or how experienced new members were with regards to MOUD delivery. Because participants’ responses were anonymous, we were not able to link participants’ responses across survey administrations. Some survey items were developed specifically for the project and have not been validated. Our findings are limited to provider perceptions and do not examine MOUD outcomes. Although provider perceptions are a consistent barrier to MOUD, future work should examine how changes in perceptions impact rates of MOUD receipt and patient outcomes.

## Conclusions

Despite these limitations, to our knowledge, this is the first report to examine changes in factors related to implementation of MOUD in a national initiative to improve access to MOUD in non-traditional settings. To combat the ongoing opioid epidemic, increasing treatment in non-SUD specialty clinics is imperative, as less than half of individuals with OUD will seek SUD treatment. With few exceptions, providers’ perspectives on MOUD were consistently positive, as was their perceived knowledge of OUD, comfort and effectiveness with working with patients who use opioids, availability of support from colleagues to deliver MOUD and satisfaction in providing such care. These perceptions remained stable over time, though the belief that MOUD fills important gaps in clinical care decreased over time. Providers endorsed several important barriers, including deficits in leadership culture, behaviors, and measurement of care, time constraints, and delays in credentialing/privileging processes to prescribe buprenorphine. Overall, our findings indicate additional implementation strategies are likely necessary to improve providers’ perceptions of MOUD and change culture on the ground. Strategies that improve leaders’ prioritization and support of MOUD delivery in clinics, additional resources (e.g., reduced panel size or care managers dedicated to implementation teams) to address time constraints associated with delivering MOUD, and streamlining credentialing and privileging processes may be necessary to increase prescribing of MOUD in settings that do not traditionally provide this treatment.

## Supplementary Information

**Additional file 1**. Stepped Care for Opioid Use Disorder Train the Trainer initiative (SCOUTT) Survey Description of data: 43-Item SCOUTT Survey Instrument developed based on review of published studies examining provider- and systems-level barriers and facilitators to providing MOUD.

**Additional file 2**. 43-Item Survey Organized into 3 Content DomainsDescription of data: SCOUTT Survey Instrument Organized by CFIR Domains including inner setting, intervention characteristics, and individual characteristics to measure additional aspects of implementation.

## Data Availability

The datasets used and/or analyzed during the current study are available from the corresponding author on reasonable request.

## Abbreviations

CESATE: Center of Excellence in Substance Addiction Treatment and Education
CFIR: Consolidated Framework for Implementation Research
DDPPQ: Drug and Drug Problems Perception Questionnaire
DEA: Drug Enforcement Administration
HSR&D: Health Services Research & Development
MOUD: Medication treatment of opioid use disorder
OMHSP: Office of Mental Health and Suicide Prevention
ORCA: Organizational Readiness to Change Assessment
OUD: Opioid use disorder
PARCKA: Program for Addiction Research, Clinical Care, Knowledge and Advocacy
QUERI: Quality Enhancement Research Initiative
SCOUTT: Stepped Care for Opioid Use Disorder Train-the-Trainer
SUD: Substance use disorder
VA: Veterans Affairs
