# Supplementary material for: Clinical leaders and providers’ perspectives on delivering medications for the treatment of opioid use disorder in Veteran Affairs’ facilities
Source: Addict Sci Clin Pract. 2021 Sep 6;16:55. doi: 10.1186/s13722-021-00263-5 (PMC8419813; doi:10.1186/s13722-021-00263-5)
Supplement: Supplementary file 1 — Additional file 1. Stepped Care for Opioid Use Disorder Train the Trainer initiative (SCOUTT) Survey Description of data: 43-Item SCOUTT Survey Instrument developed based on review of published studies examining provider- and systems-level barriers and facilitators to providing MOUD. [file 13722_2021_263_MOESM1_ESM.pdf]

VA Stepped Care for Opioid Use Disorder Train-the-Trainer (SCOUTT) Initiative - Follow Up Survey

Thank you for agreeing to take part in this survey!

As part of a larger evaluation of the Stepped Care for Opioid Use Disorder Train-the-Trainer (SCOUTT) roll out across the VA, we would like to learn more about the providers involved in SCOUTT and their perspectives on treating opioid use disorder (OUD) over the first year of implementation. Participation is voluntary and you have the option to not respond to any questions that you choose. The survey should take 7-10 minutes to complete. Your responses are anonymous, and will be shared only in aggregate to imorove the quality of Veterans' care.

Definitions of acronyms in this survey:

- **OUD = Opioid Use Disorder**
- **SUD = Substance Use Disorder**
- **SCOUTT = Stepped Care for Opioid Use Disorder Train-the-Trainer Initiative**
- **M-OUD = Medication treatment for opioid use disorder is the use of medications (e.g., buprenorphine or naltrexone) in combination with medication management and behavioral therapies**

VA Stepped Care for Opioid Use Disorder Train-the-Trainer (SCOUTT) Initiative - Follow Up Survey

1. What is your age?

- ◯ 25 - 34
- ◯ 35 - 44
- ◯ 45 - 54
- ◯ 55 - 64
- ◯ 65+

2. What is your gender?

- ◯ Male
- ◯ Female

3. Years of VA practice:

- ≤ 5
- 5 - 10
- 11 - 20
- 20+

4. Total years of practice:

- ≤ 5
- 5 - 10
- 11 - 20
- 20+

5. Are you of Hispanic, Latino, or Spanish origin?

- Yes
- No

6. Which race best describes you?

- American Indian or Alaska Native
- Asian
- Native Hawaiin or Other Pacific Islander
- Black or African American
- White or Caucasian
- Multiple ethnicity or Other (please specify)

7. In which VISN do you work?

8. Please select your discipline:

- ◯ Nurse
- ◯ Nurse Practitioner
- ◯ Pharmacist
- ◯ Physician
- ◯ Physician Assistant
- ◯ Psychologist
- ◯ Social Worker
- ◯ Addiction Therapist
- ◯ Other (please specify)

[ ]

9. Please select your specialty:

- ◯ Primary Care/General Internal Medicine/Family Medicine
- ◯ Medical Specialty Care
- ◯ Mental Health
- ◯ Pain Management
- ◯ Substance Use Disorder Specialty Care
- ◯ Not Applicable (not a clinical care provider)
- ◯ Other (please specify)

[ ]

10. Please select your primary facility role:

- ◯ Pharmacist
- ◯ Prescribing Clinician
- ◯ Non-prescribing Clinician
- ◯ Leadership (defined as Clinic Director or higher level administrator)

11. Are you a prescriber?

- Yes
- No

VA Stepped Care for Opioid Use Disorder Train-the-Trainer (SCOUTT) Initiative - Follow Up Survey

12. Are you waivered by the DEA to prescribe buprenorphine?

- Yes
- No

VA Stepped Care for Opioid Use Disorder Train-the-Trainer (SCOUTT) Initiative - Follow Up Survey

13. Have you prescribed buprenorphine to treat OUD in the last 12 months?

- Yes
- No

VA Stepped Care for Opioid Use Disorder Train-the-Trainer (SCOUTT) Initiative - Follow Up Survey

14. How would you rate your experience with prescribing buprenorphine to treat OUD?

1 (No experience)	2	3	4	5 (Moderately experienced)	6	7	8	9	10 (Very experienced)

VA Stepped Care for Opioid Use Disorder Train-the-Trainer (SCOUTT) Initiative - Follow Up Survey

15. Have you prescribed naltrexone to treat OUD in the last 12 months?

- Yes
- No

VA Stepped Care for Opioid Use Disorder Train-the-Trainer (SCOUTT) Initiative - Follow Up Survey

16. How would you rate your experience with prescribing naltrexone to treat OUD?

1 (No experience)	2	3	4	5 (Moderately experienced)	6	7	8	9	10 (Very experienced)

VA Stepped Care for Opioid Use Disorder Train-the-Trainer (SCOUTT) Initiative - Follow Up Survey

17. On a scale from 1 (not prepared at all) to 10 (very prepared), how prepared are you to:

	1	2	3	4	5	6	7	8	9	10	N/A
assess patients for OUD											
diagnose OUD											
educate patients about treatment options for OUD											

18. On a scale from 1 (not prepared at all) to 10 (very prepared), how prepared is your clinic to:

	1	2	3	4	5	6	7	8	9	10
provide medications for treatment of OUD										

VA Stepped Care for Opioid Use Disorder Train-the-Trainer (SCOUTT) Initiative - Follow Up Survey

19. Please indicate your level of agreement with the following statements about your clinic’s functioning:

	Strongly Disagree	Disagree	Neither Agree nor Disagree	Agree	Strongly Agree	N/A
Overall, our team has done its work well this last month	○	○	○	○	○	○
In general, SCOUTT implementation clinics deliver high quality M-OUD services	○	○	○	○	○	○
Members of our team depend on each other to deliver M-OUD services	○	○	○	○	○	○
We are a team of people with a shared task – not a collection of individuals who have their own particular jobs to do	○	○	○	○	○	○
In general, members of our team would agree that we have worked well as a team this past month	○	○	○	○	○	○
Overall, the different patient-related jobs and activities that everyone does on this team fit together very well	○	○	○	○	○	○

20. What challenges have you faced in your role as a SCOUTT implementation clinic provider?

VA Stepped Care for Opioid Use Disorder Train-the-Trainer (SCOUTT) Initiative - Follow Up Survey

21. To what extent have your clinic’s practices changed as a result of the SCOUTT roll-out across the VA?

- Not at all
- Slightly
- Moderately
- Quite a Bit
- Extremely

VA Stepped Care for Opioid Use Disorder Train-the-Trainer (SCOUTT) Initiative - Follow Up Survey

22. Please specify how you clinic's practices have changed?

VA Stepped Care for Opioid Use Disorder Train-the-Trainer (SCOUTT) Initiative - Follow Up Survey

23. Delivering medications (buprenorphine or naltrexone) to treat OUD in my clinic:

	Strongly Disagree	Disagree	Neither Agree nor Disagree	Agree	Strongly Agree
is important					
will save lives					
is time consuming					
detracts from my clinical responsibilities					
is more dangerous than management of other chronic diseases					
can be done successfully					

24. Delivering medications (buprenorphine or naltrexone) to treat OUD in my clinic:

	Strongly Disagree	Disagree	Neither Agree nor Disagree	Agree	Strongly Agree
is supported by randomized clinical trials or other scientific evidence	◯	◯	◯	◯	◯
conforms to the opinions of clinical experts in my clinic	◯	◯	◯	◯	◯
is consistent with clinical practices that have been accepted by VA patients	◯	◯	◯	◯	◯
fills an important gap in the care my clinic provides	◯	◯	◯	◯	◯
can be integrated into my clinic’s procedures and workflow	◯	◯	◯	◯	◯
is compatible with the care provided by my clinic	◯	◯	◯	◯	◯

25. Please indicate your level of agreement or disagreement with the following statements:

	Strongly Disagree	Disagree	Neither Agree nor Disagree	Agree	Strongly Agree
The risk of patients diverting these medications is too high	◯	◯	◯	◯	◯
Providers in my clinic want to prescribe buprenorphine or naltrexone	◯	◯	◯	◯	◯

VA Stepped Care for Opioid Use Disorder Train-the-Trainer (SCOUTT) Initiative - Follow Up Survey

26. Please indicate your level of agreement or disagreement with each of the following statements about working with people who use illicit or legal opioids in a non-therapeutic way:

	1 (Strongly Agree)	2	3	4 (Neither Agree nor Disagree)	5	6	7 (Strongly Disagree)
I feel I have a working knowledge of opioids and opioid-related problems.	◯	◯	◯	◯	◯	◯	◯
I feel I know enough about the causes of drug problems to carry out my role when working with opioid users.	◯	◯	◯	◯	◯	◯	◯
I feel I know enough about the physical effects of drug use to carry out my role when working with opioid users.	◯	◯	◯	◯	◯	◯	◯
I feel I know enough about the psychological effects of drugs to carry out my role when working with opioid users.	◯	◯	◯	◯	◯	◯	◯
I feel I know enough about the factors which put people at risk of developing drug problems to carry out my role when working with opioid users.	◯	◯	◯	◯	◯	◯	◯

27. Please indicate your level of agreement or disagreement with each of the following statements about working with people who use illicit or legal opioids in a non-therapeutic way:

	1 (Strongly Agree)	2	3	4 (Neither Agree nor Disagree)	5	6	7 (Strongly Disagree)
I feel I know how to counsel opioid users over the long-term.	◯	◯	◯	◯	◯	◯	◯
I feel I can appropriately advise my patients about opioids and their effects.	◯	◯	◯	◯	◯	◯	◯
I feel I have the right to ask patients questions about their opioid use when necessary.	◯	◯	◯	◯	◯	◯	◯
I feel I have the right to ask patients for any information that is relevant to their opioid-related problems.	◯	◯	◯	◯	◯	◯	◯

VA Stepped Care for Opioid Use Disorder Train-the-Trainer (SCOUTT) Initiative - Follow Up Survey

28. Please indicate your level of agreement or disagreement with each of the following statements about working with people who use illicit or legal opioids in a non-therapeutic way:

	1 (Strongly Agree)	2	3	4 (Neither Agree nor Disagree)	5	6	7 (Strongly Disagree)
If I felt the need when working with opioid users, I could easily find someone with whom I could discuss any personal difficulties that I might encounter.	◯	◯	◯	◯	◯	◯	◯
If I felt the need when working with opioid users, I could easily find someone who would help me clarify my professional responsibilities.	◯	◯	◯	◯	◯	◯	◯
If I felt the need, I could easily find someone who would be able to help me formulate the best approach for an opioid user.	◯	◯	◯	◯	◯	◯	◯
I feel that there is little I can do to help opioid users.	◯	◯	◯	◯	◯	◯	◯
I feel I am able to work with opioid users as well as other patient groups.	◯	◯	◯	◯	◯	◯	◯

29. Please indicate your level of agreement or disagreement with each of the following statements about working with people who use illicit or legal opioids in a non-therapeutic way:

	1 (Strongly Agree)	2	3	4 (Neither Agree nor Disagree)	5	6	7 (Strongly Disagree)
In general, I have less respect for opioid users than for most other patients I work with.	◯	◯	◯	◯	◯	◯	◯
In general, one can get satisfaction from working with opioid users.	◯	◯	◯	◯	◯	◯	◯
In general, it is rewarding to work with opioid users.	◯	◯	◯	◯	◯	◯	◯
In general, I feel I can understand opioid users.	◯	◯	◯	◯	◯	◯	◯
I often feel uncomfortable when working with opioid users.	◯	◯	◯	◯	◯	◯	◯
All in all I am inclined to feel I am a failure with opioid users.	◯	◯	◯	◯	◯	◯	◯

30. How helpful would the following resources be to manage opioid use disorder in your clinic?

	Not at All	Slightly	Moderately	Quite a Bit	Extremely	Not applicable (Resource is available)
Consultation with experts	○	○	○	○	○	○
Continuing education/skills training	○	○	○	○	○	○
More time with patients to discuss medications for OUD	○	○	○	○	○	○
Same-day access to M-OUD services	○	○	○	○	○	○
Assistance with identifying patients potentially at risk of OUD	○	○	○	○	○	○
Assistance with monitoring urine toxicology screens	○	○	○	○	○	○
Mental health or psychosocial support services	○	○	○	○	○	○
Support for credentialing/privileging waivered buprenorphine prescribers	○	○	○	○	○	○

Other (please specify)

VA Stepped Care for Opioid Use Disorder Train-the-Trainer (SCOUTT) Initiative - Follow Up Survey

Please indicate your level of agreement or disagreement with the following statements:

31. Senior leadership/clinical management in your organization:

	Strongly Disagree	Disagree	Neither Agree nor Disagree	Agree	Strongly Agree	Don't Know/Not Applicable
reward clinical innovation and creativity to improve patient care	○	○	○	○	○	○
solicit opinions of clinical staff regarding decisions about patient care	○	○	○	○	○	○
seek ways to improve patient education and increase patient participation in treatment	○	○	○	○	○	○
provide effective management for continuous improvement of patient care	○	○	○	○	○	○
clearly define areas of responsibility and authority for clinical managers and staff	○	○	○	○	○	○

32. Senior leadership/clinical management in your organization:

	Strongly Disagree	Disagree	Neither Agree nor Disagree	Agree	Strongly Agree	Don't Know/Not Applicable
promote team building to solve clinical care problems						
promote communication among clinical services and units						
provide staff with information on VA performance measures and guidelines						
establish clear goals for patient care processes and outcomes						
provide staff members with feedback/data on effects of clinical decisions						
hold staff members accountable for achieving results						

VA Stepped Care for Opioid Use Disorder Train-the-Trainer (SCOUTT) Initiative - Follow Up Survey

Please indicate your level of agreement or disagreement with the following statements:

33. Staff members in your clinic:

	Strongly Disagree	Disagree	Neither Agree nor Disagree	Agree	Strongly Agree	Don't Know/Not Applicable
have a sense of personal responsibility for improving patient care and outcomes						
cooperate to maintain and improve effectiveness of patient care						
are willing to innovate and/or experiment to improve clinical procedures						
are receptive to change in clinical processes						

34. Opinion leaders (respected/influential colleagues) in your clinic:

	Strongly Disagree	Disagree	Neither Agree nor Disagree	Agree	Strongly Agree	Don't Know/Not Applicable
believe that the current practice patterns can be improved						
encourage and support changes in practice patterns to improve patient care						
are willing to try new clinical protocols						
work cooperatively with senior leadership/clinical management to make appropriate changes						

VA Stepped Care for Opioid Use Disorder Train-the-Trainer (SCOUTT) Initiative - Follow Up Survey

35. Please estimate the amount of time you spend identifying and/or talking to a patient who needs treatment for OUD? **(minutes per patient)**

36. Before SCOUTT, did you provide OUD-related care?

- Yes
- No

VA Stepped Care for Opioid Use Disorder Train-the-Trainer (SCOUTT) Initiative - Follow Up Survey

37. How has SCOUTT affected the amount of time you spend providing OUD-related care?

- Reduced
- Increased
- Don't Know

VA Stepped Care for Opioid Use Disorder Train-the-Trainer (SCOUTT) Initiative - Follow Up Survey

38. Please estimate how much time is being **SAVED** per patient per visit?

- 0-10 minutes
- 11-20 minutes
- 21-30 minutes
- 31-60 minutes
- 60+ minutes
- Don't Know

VA Stepped Care for Opioid Use Disorder Train-the-Trainer (SCOUTT) Initiative - Follow Up Survey

39. Please estimate how much **MORE** time is being spent per patient per visit?

- 0-10 minutes
- 11-20 minutes
- 21-30 minutes
- 31-60 minutes
- 60+ minutes
- Don't Know

VA Stepped Care for Opioid Use Disorder Train-the-Trainer (SCOUTT) Initiative - Follow Up Survey

40. How much time do you spend providing OUD-related care **per patient per visit?**

- 0-10 minutes
- 11-20 minutes
- 21-30 minutes
- 31-60 minutes
- 60+ minutes
- Don't Know

VA Stepped Care for Opioid Use Disorder Train-the-Trainer (SCOUTT) Initiative - Follow Up Survey

41. How many providers at your facility have you trained?

42. Please estimate the amount of time you spend training providers at your facility? **(minutes per provider)**

43. Please describe your experience with SCOUTT facilitation activities?

For example: SCOUTT monthly calls, SharePoint site, site visits, working with the facilitation team (Dr. Princess Ackland, Dr. Hildi Hagedorn, Marie Kenny, Dr. Amanda Midboe, Dr. Adam Gordon and/or Dr. Karen Drexler)
