# Supplementary material for: Clinical leaders and providers’ perspectives on delivering medications for the treatment of opioid use disorder in Veteran Affairs’ facilities
Source: Addict Sci Clin Pract. 2021 Sep 6;16:55. doi: 10.1186/s13722-021-00263-5 (PMC8419813; doi:10.1186/s13722-021-00263-5)
Supplement: Supplementary file 2 — Additional file 2. 43-Item Survey Organized into 3 Content DomainsDescription of data: SCOUTT Survey Instrument Organized by CFIR Domains including inner setting, intervention characteristics, and individual characteristics to measure additional aspects of implementation. [file 13722_2021_263_MOESM2_ESM.docx]

Supplemental Table 1. 43-Item Survey Organized into 3 Content Domains

| CFIR  Domain | Survey Content Domains |
| --- | --- |
|  | 1. Background information |
| Intervention Characteristics | 1. Beliefs about medication treatment for opioid use disorder |
| Inner Setting | 1. Beliefs about staff and leadership culture, behavior and organizational receptivity to medication treatment for opioid use disorder |
| Characteristics of Individuals | 1. Knowledge and beliefs about providers role, comfort, and satisfaction delivering medication treatment for opioid use disorder |
